# Signal peptidase complex catalytic subunit *SEC11A* upregulation is a biomarker of poor prognosis in patients with head and neck squamous cell carcinoma

**DOI:** 10.1371/journal.pone.0269166

**Published:** 2022-06-02

**Authors:** Chunmei Hu, Jiangang Fan, Gang He, Chuan Dong, Shijie Zhou, Yun Zheng

**Affiliations:** 1 Department of Otorhinolaryngology Head & Neck Surgery, West China Hospital, Sichuan University, Chengdu, Sichuan, China; 2 Department of Otolaryngology Head and Neck Surgery, Sichuan Provincial People’s Hospital, University of Electronic Science and Technology of China, Chengdu, Sichuan, China; 3 State Key Laboratory of Biotherapy and Cancer Center, West China Hospital, Sichuan University and Collaborative Innovation Center of Biotherapy, Chengdu, Sichuan, China; University of California, San Francisco, UNITED STATES

## Abstract

In the current study, we aimed to investigate the expression of the five microsomal signal peptidase complex (SPC) subunit genes (*SEC11A*, *SEC11C*, *SPCS1*, *SPCS2*, and *SPCS3*) in head and neck squamous cell carcinoma (HNSC) and to explore their prognostic value. Data from the HNSC subset of The Cancer Genome Atlas (TCGA) and one previous single-cell RNA-seq dataset was used. Subgroup analysis was conducted in tumors from different anatomic sites. Gene set enrichment analysis (GSEA), and immune cell infiltration analysis were performed to check the influence of SEC11A on the tumor microenvironment. Among the genes significantly upregulated in the tumor group, only *SEC11A* expression (as a continuous variable) is independently associated with poorer progression-free survival (PFS) (HR: 2.075, 95%CI: 1.447–2.977, *p*<0.001) and disease-specific survival (DSS) (HR: 2.023, 95%CI: 1.284–3.187, *p* = 0.002). Subgroup analysis confirmed the prognostic value in tumors from three anatomic origins, including laryngeal squamous cell carcinoma, oral cavity-related squamous cell carcinoma, and oropharynx-related squamous cell carcinoma. *SEC11A* is expressed in all subtypes of cells in the tumor microenvironment. Its expression showed a moderate positive correlation with its gene-level copy number (Pearson’s r = 0.53, *p*<0.001). *SEC11A* expression was negatively correlated with CD8+ T cells and B cells, but was positively correlated with cancer-associated fibroblast and myeloid-derived suppressor cells (MDSCs) in the tumor microenvironment. In summary, *SEC11A* upregulation is a result of gene amplification in head and neck squamous cell carcinoma. Its upregulation might serve as an independent prognostic biomarker and a predictor of the infiltration of certain types of immune cells.

## Introduction

The microsomal signal peptidase complex (SPC), which is an essential membrane component in the endoplasmic reticulum (ER), has five subunits, including SEC11A, SEC11C, SPCS1, SPCS2, and SPCS3 [1]. Functionally, SPC removes signal peptides (SPs) from a series of secretory pre-proteins with high specificity [1]. Among the subunits, SEC11A has been characterized as a penitential oncogene and a valuable prognostic biomarker in multiple types of solid tumors. *SEC11A* upregulation is associated with worse survival of gastric cancer [2]. It facilitates gastric cancer cell growth *in vitro* and *in vivo* by promoting the expression of the secretion of TGF-α secretion [2]. SEC11A can promote the phosphorylation of epidermal growth factor receptor (EGFR) and the downstream extracellular signal-regulated kinase (ERK) and protein kinase B (Akt) in colorectal cancer cells [3]. Its expression serves as an independent prognostic biomarker for patients with CRC [3], basal-Like bladder cancer [4] and esophageal squamous cell carcinoma [5]. One recent study found that inhibiting *SEC11A* can reduce proliferation, migration, and invasion and induce apoptosis of tongue squamous cell carcinoma [6], suggesting a potential oncogenic role of this gene in head and neck squamous cell carcinoma.

The dysregulation of the other SPC subunits was also observed in some tumors. *SPCS1* expression can be affected by FOXM1-modulated gene promoter methylation in head and neck squamous cell carcinoma [7]. *SPCS2* has a significant diagnostic value for hepatocellular carcinoma (HCC) [8]. *SPCS3* expression can be used as a component of a risk score model to predict the survival of patients with pancreatic adenocarcinoma [9].

Head and neck squamous cell carcinoma is a group of heterogenous carcinomas derived from different anatomic sites. Most patients are diagnosed in advanced stages (stage III and IV). More than half of the cases suffer locally advanced disease recurrence even after curative therapy. The median overall survival for the cases with recurrent or metastatic tumors is shorter than one year [10]. Therefore, it is necessary to explore reliable prognostic biomarkers to support better disease management.

In the current study, we aimed to investigate the expression profile of the SPC subunit genes in head and neck squamous cell carcinoma and explore their prognostic value. Subgroup analysis was conducted in tumors from different anatomic sites. Gene set enrichment analysis (GSEA), and immune cell infiltration analysis were performed to check the influence of SEC11A on the tumor microenvironment.

## Materials and methods

This study is a secondary bioinformatic analysis based on online open datasets. No primary samples were collected in the current study. Therefore, ethical approval is not required. Data in the databases are fully anonymous. Informed consent was appropriately handled by the original studies relevant to the databases.

### Data extraction

Data extraction was performed using the UCSC Xena browser [11]. The five SPC subunit gene expression, gene-level methylation, gene-level copy number data, clinicopathological and survival data were extracted from the HNSC subset in The Cancer Genome Atlas (TCGA)-pan-cancer database, following the methods we described previously [12]. Single-cell RNA-seq data of *SEC11A* expression from more than 5000 cells from head and neck squamous cell carcinomas were extracted from Puram 2017 dataset [13].

### Cell culture and immunofluorescent staining

SCC9 and TU212 cell culture and immunofluorescent staining were conducted as we previously described [14]. For immunofluorescent staining, cells grown on coverslips were fixed, permeabilized, and blocked. Then, coverslips were incubated with anti-SEC11A (1:100, A10552, ABclonal, Wuhan, China) at 4 ℃ overnight. Then, the coverslips were washed and incubated with a secondary antibody (anti-rabbit IgG (H+L), F(ab’)2 Fragment, Alexa Fluor 647 Conjugate) (Cell Signaling, Danvers, MA, USA) for 1 h at 37°C. Then, the coverslips were washed and mounted with a mounting medium containing DAPI. IF images were captured using a fluorescence microscopy (Nikon, 90i, Japan).

### Immunohistochemistry (IHC) staining

IHC was performed using a commercial human paraffin-embedded head and neck squamous cell carcinoma tissue array and anti-SEC11A (1:200, A10552, ABclonal, Wuhan, China), on a BOND-III Automated IHC Stainer (Leica Microsystems GmbH, Wetzlar, Germany).

### Gene Set Enrichment Analysis (GSEA)

GSEA was performed among patients with primary head and neck squamous cell carcinoma. Gene set enrichment was compared between the high (top 50%) and low (bottom 50%) *SEC11A* expression groups using the GSEA software (v 4.1.0). The h.all.v7.5.sytmbols.gmt in Molecular Signatures Database (MSigDB) was selected as the reference gene set. Only the gene set with nominal (Nom) *p* <0.05 and false discovery rate (FDR) q <0.05 were included.

### Statistical analysis

Survival curves (both progression-free survival, PFS and disease-specific survival, DSS) were estimated using the Kaplan-Meier method and median gene expression. Univariate and multivariate analysis by Cox proportional hazards model was performed to assess the independent prognostic value of *SEC11A* expression as a continuous variable. Only the variates with *p* value <0.1 were included in the multivariate analysis. Log-rank test was used to compare the curves and calculate the *p* values. Correlation analysis was performed by calculating the Pearson’s correlation coefficients. *p*<0.05 was considered statistically significant.

## Results

### SEC11A expression independently predicts unfavorable survival of patients with head and neck squamous cell carcinoma

**Fig 1:**
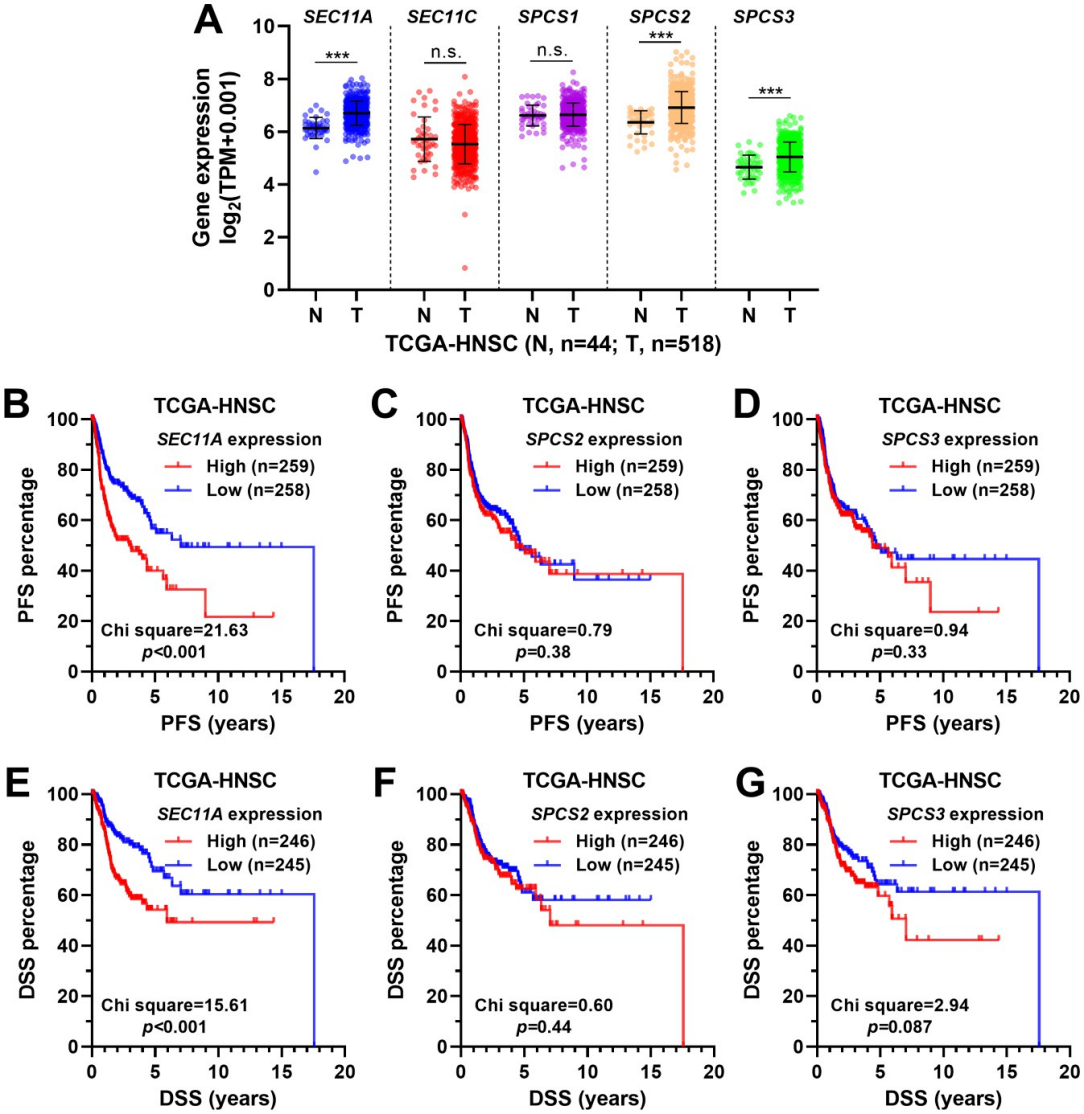
The expression profile of SPC subunit genes and their association with survival. **A.** The expression of the five SPC subunit genes between primary head and neck squamous cell carcinoma tissues (n = 518) and tumor-adjacent normal tissues (n = 44), TCGA-HNSC. **B-G.** K-M survival analysis was performed to compare the differences in PFS (B-D) and DSS (E-G). Patients were grouped by median expression of *SEC11A* (B and E), *SPCS2* (C and F) and *SPCS3* (D and G) expression. Log-rank test was used to compare the curves and calculate the *p* values.

We extracted RNA-seq data from primary head and neck squamous cell carcinoma (n = 518) and tumor-adjacent normal tissues (n = 44) and compared the expression profile of the five SPC subunit genes. Results showed that *SEC11A*, *SPCS2* and *SPCS3* were significantly upregulated in the tumor group than in the normal group (Fig 1A). K-M survival analysis revealed that patients with high (higher 50%) *SEC11A* expression had significantly shorter PFS and DSS compared to the low expression (lower 50%) group (Fig 1B and 1E). However, these trends were not observed between groups by median *SPCS2* or *SPCS3* separation (Fig 1C, 1D, 1F and 1G).

The clinicopathological characteristics of patients with high and low *SEC11A* expression groups were compared (Table 1). No significant difference was observed in histological grade, margin status, AJCC stage, gender and age between the two groups (*p*>0.05, Table 1). However, the high *SEC11A* expression group had a significantly higher ratio of PFS events (117/259, 22.6% vs. 80/259, 15.4%, *p* = 0.001) and DSS events (80/246, 16.3% vs. 50/246, 10.2%, *p* = 0.003) compared to the low *SEC11A* expression group (Table 1).

**Table 1:** Comparison of clinicopathological characteristics between high and low *SEC11A* expression groups.

Characteristic	Group 1 (High *SEC11A*)	Group 2 (Low *SEC11A*)	*p*-value	Statistic	Method
**Number**	259	259			
**Histological grade, n (%)**			0.369	4.28	Chisq. test
G1	23 (4.5%)	38 (7.4%)			
G2	157 (30.5%)	147 (28.6%)			
G3	64 (12.5%)	60 (11.7%)			
G4	3 (0.6%)	4 (0.8%)			
Gx	9 (1.8%)	9 (1.8%)			
**Margin status, n (%)**			0.115	4.33	Chisq. test
Close	27 (5.9%)	21 (4.6%)			
Negative	167 (36.5%)	183 (40%)			
Positive	36 (7.9%)	23 (5%)			
**AJCC stage, n (%)**			0.066	7.2	Chisq. test
Stage I	9 (2%)	18 (4.1%)			
Stage II	29 (6.5%)	41 (9.3%)			
Stage III	40 (9%)	40 (9%)			
Stage IV	145 (32.7%)	121 (27.3%)			
**Gender, n (%)**			0.765	0.09	Chisq. test
Female	70 (13.5%)	66 (12.7%)			
Male	189 (36.5%)	193 (37.3%)			
**Age, median (IQR)**	61 (53, 68)	60.5 (53, 69)	0.999	33407.5	Wilcoxon
**PFS event, n (%)**			0.001	10.62	Chisq. test
No occurrence of NTE	142 (27.4%)	179 (34.6%)			
Occurrence of a NTE	117 (22.6%)	80 (15.4%)			
**DSS event, n (%)**			0.003	8.79	Chisq. test
Alive	166 (33.7%)	196 (39.8%)			
Death from the diagnosed cancer	80 (16.3%)	50 (10.2%)			

Gx: grade cannot be assessed; AJCC: American Joint Committee on Cancer; IQR: interquartile range; margin status: positive (tumor ≤1 mm from the margin), close (between 1 and 5 mm). NTE: new tumor event.

Therefore, we further explored whether *SEC11A* expression serves as an independent prognostic biomarker. Univariate and multivariate analysis confirmed that *SEC11A* expression (as a continuous variable) is independently associated with poorer PFS (HR: 2.075, 95%CI: 1.447–2.977, *p*<0.001), after adjustment for histological grade, margin status, and AJCC stage (Table 2). Additionally, *SEC11A* expression also independently predicted poorer DSS (HR: 2.023, 95%CI: 1.284–3.187, *p* = 0.002), after adjusting histological grade, margin status, and AJCC stage (Table 3).

**Table 2:** Univariate and multivariate analysis for PFS.

Characteristics	Total (N)	Univariate analysis		Multivariate analysis	
		Hazard ratio (95% CI)	*p-*value	Hazard ratio (95% CI)	*p-*value
***SEC11A***	517	2.173 (1.584–2.982)	**<0.001**	2.075 (1.447–2.977)	**<0.001**
**Histological grade**	513				
G3	124	Reference			
G1	61	0.768 (0.456–1.293)	0.320		
G2	303	1.140 (0.815–1.593)	0.445		
G4	7	0.658 (0.160–2.711)	0.562		
GX	18	0.238 (0.058–0.978)	**0.046**		
**Margin status**	456				
Negative	349	Reference			
Positive	59	2.210 (1.532–3.188)	**<0.001**	1.891 (1.280–2.794)	**0.001**
Close	48	1.722 (1.079–2.747)	**0.023**	1.868 (1.162–3.004)	**0.010**
**AJCC stage**	442				
Stage I	27	Reference			
Stage IV	266	1.498 (0.760–2.953)	0.243	1.488 (0.754–2.937)	0.252
Stage III	80	0.898 (0.417–1.933)	0.783	0.959 (0.443–2.075)	0.915
Stage II	69	0.675 (0.303–1.504)	0.336	0.698 (0.310–1.574)	0.387
**Age**	517	1.008 (0.995–1.021)	0.226		
**Gender**	517				
Male	381	Reference			
Female	136	0.986 (0.715–1.360)	0.931		

**Table 3:** Univariate and multivariate analysis for DSS.

Characteristics	Total (N)	Univariate analysis		Multivariate analysis	
		Hazard ratio (95% CI)	*p*-value	Hazard ratio (95% CI)	*p*-value
***SEC11A***	491	2.364 (1.599–3.494)	**<0.001**	2.023 (1.284–3.187)	**0.002**
**Histological grade**	488				
G3	118	Reference			
G1	58	0.656 (0.341–1.261)	0.206		
G2	291	1.042 (0.700–1.553)	0.838		
G4	7	0.000 (0.000-Inf)	0.993		
GX	14	0.236 (0.032–1.721)	0.154		
**Margin status**	433				
Negative	330	Reference			
Positive	58	2.718 (1.784–4.141)	**<0.001**	2.318 (1.474–3.644)	**<0.001**
Close	45	1.737 (0.960–3.144)	0.068	1.884 (1.031–3.444)	**0.040**
**AJCC stage**	420				
Stage I	27	Reference			
Stage IV	254	3.144 (0.993–9.950)	0.051	3.141 (0.992–9.951)	0.052
Stage III	75	1.777 (0.514–6.141)	0.363	1.895 (0.546–6.580)	0.314
Stage II	64	1.395 (0.389–5.003)	0.610	1.446 (0.396–5.278)	0.576
**Age**	491	1.009 (0.993–1.025)	0.272		
**Gender**	491				
Male	366	Reference			
Female	125	1.063 (0.715–1.579)	0.763		

### Subgroup analysis of PFS and DSS in laryngeal, oral cavity related, and oropharynx related squamous cell carcinoma

**Fig 2:**
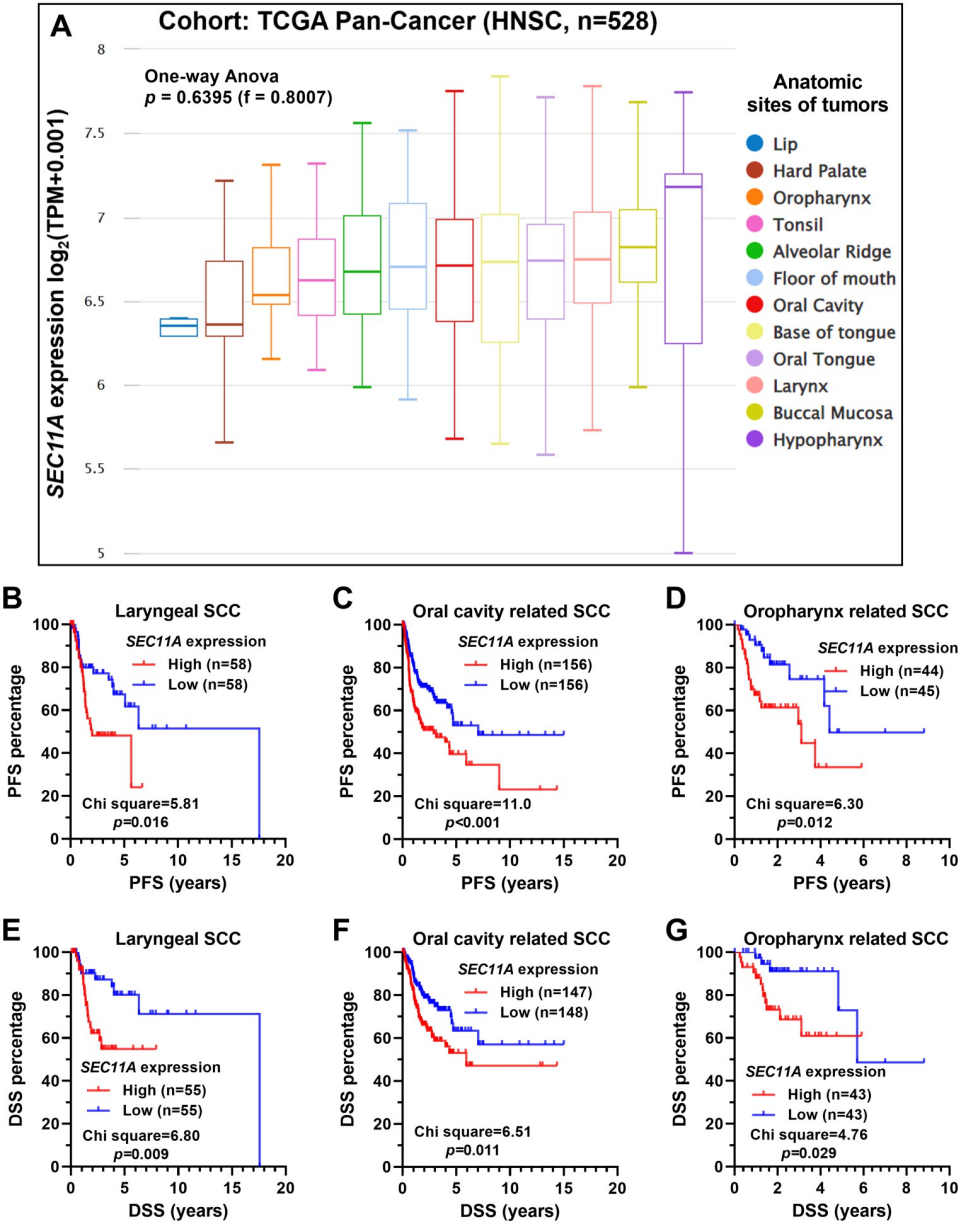
Subgroup analysis of PFS and DSS in laryngeal, oral cavity related, and oropharynx related squamous cell carcinoma. **A.** The expression of *SEC11A* in tumors from different anatomic sites. **B-G.** K-M survival analysis was performed to compare the differences in PFS (B-D) and DSS (E-G) in patients grouped by anatomic sites, including laryngeal squamous cell carcinoma (B and E), oral cavity related squamous cell carcinoma (C and F) and oropharynx related squamous cell carcinoma (D and G). SCC, squamous cell carcinoma. Log-rank test was used to compare the curves and calculate the *p* values.

Since head and neck squamous cell carcinoma is a group of heterogeneous tumors with different anatomic origins, we compared *SEC11A* expression among different anatomic subgroups (Fig 2A). One-way ANOVA did not detect significant differences among the subgroups (*p* = 0.64, Fig 2A). Then, according to the anatomic origins, we divided the tumor cases into three categories, including laryngeal squamous cell carcinoma, oral cavity related squamous cell carcinoma (tumors from alveolar ridge, buccal mucosa, floor of mouth, hard palate, lip, oral cavity, and oral tongue), and oropharynx related squamous cell carcinoma (tumors from the base of tongue, hypopharynx, oropharynx and tonsil). K-M survival analysis showed that high (higher 50%) *SEC11A* expression was consistently associated with significantly shorter PFS and DSS in these three subgroups (Fig 2B–2G). Since HPV positive and HPV negative head and neck squamous cell carcinoma tumors have distinct molecular profiles and biological behaviors [15], we further assessed whether *SEC11A* expression is consistently associated with PFS and DSS in these subgroups. In the cases with known HPV16 infection status in TCGA-HNSC. K-M survival curves suggested that the high *SEC11A* expression group might have worse survival (S1A–S1D Fig). However, the sample sizes of these subgroups are relatively small (38 HPV16 positive cases and 73 HPV16 negative cases). Statistical analysis only confirmed a significant difference in HPV negative subgroup in terms of DSS (S1D Fig).

### Analysis of *SEC11A* expression in the tumor microenvironment

**Fig 3:**
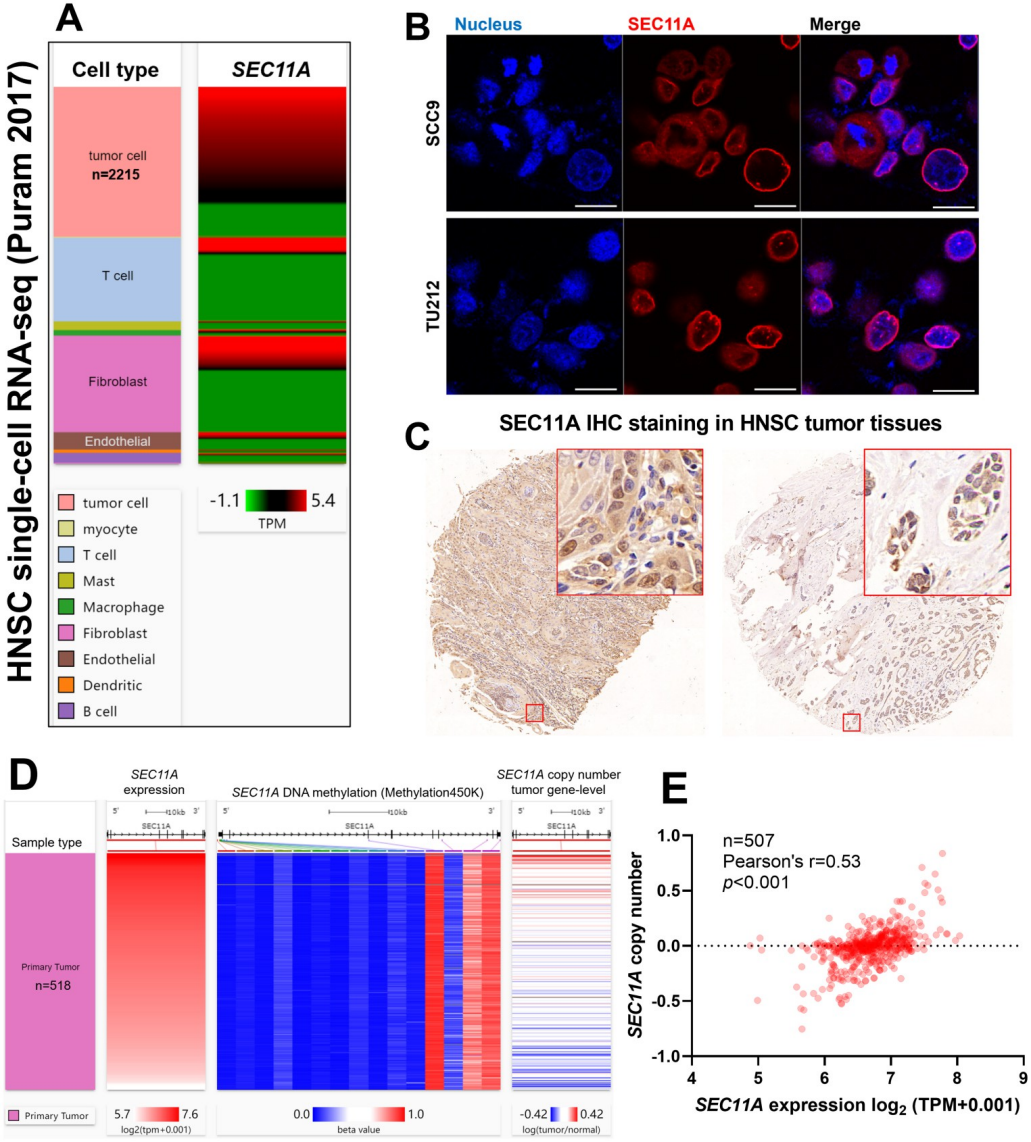
Analysis of *SEC11A* expression in the tumor microenvironment. **A.** The expression profile of *SEC11A* in different cell types in Puram 2017’s single-cell dataset. **B.** Immunofluorescent staining was conducted to show SEC11A localization in SCC9 and TU212 cells. **C.** Representative images of SEC11A protein expression (IHC staining) in primary head and neck squamous cell carcinoma tissues. **D.** A heatmap showing the correlation among *SEC11A* expression, *SEC11A* gene locus methylation, and gene-level *SEC11A* copy number in primary head and neck squamous cell carcinoma cases in TCGA. **E.** A plot chart showing the spearman correlation between *SEC11A* gene expression and gene-level copy number.

Using single-cell RNA-seq data (Puram 2017) from one previous publication [13], we checked *SEC11A* expression in the tumor microenvironment. Results confirmed its expression in all subtypes of cells, such as tumor cells, T cells, fibroblast, endothelial cells and B cells (Fig 3A). By performing immunofluorescent staining in a tongue squamous cell carcinoma cell line (SCC9) and a laryngeal squamous cell carcinoma cell line (TU212), we observed SEC11A expression in the nucleus membrane and cytoplasm (Fig 3B). Via IHC staining of SEC11A in a commercial head and neck squamous cell carcinoma tissue array, we confirmed a similar subcellular location of SEC11A in immunofluorescent staining (Fig 3C). To explore the mechanisms leading to *SEC11A* dysregulation, we checked the methylation status of CpG sites within the *SEC11A* gene locus in Illumina HumanMethylation450 BeadChip and gene-level copy number in TCGA-HNSC (Fig 3D). No significant correlation was observed between *SEC11A* expression and the methylation status of the CpG sites. However, *SEC11A* expression showed a moderate positive correlation with its copy number (Pearson’s r = 0.53, *p*<0.001, Fig 3E).

### *SEC11A* expression might predict the infiltration of certain types of immune cells in the tumor microenvironment

**Fig 4:**
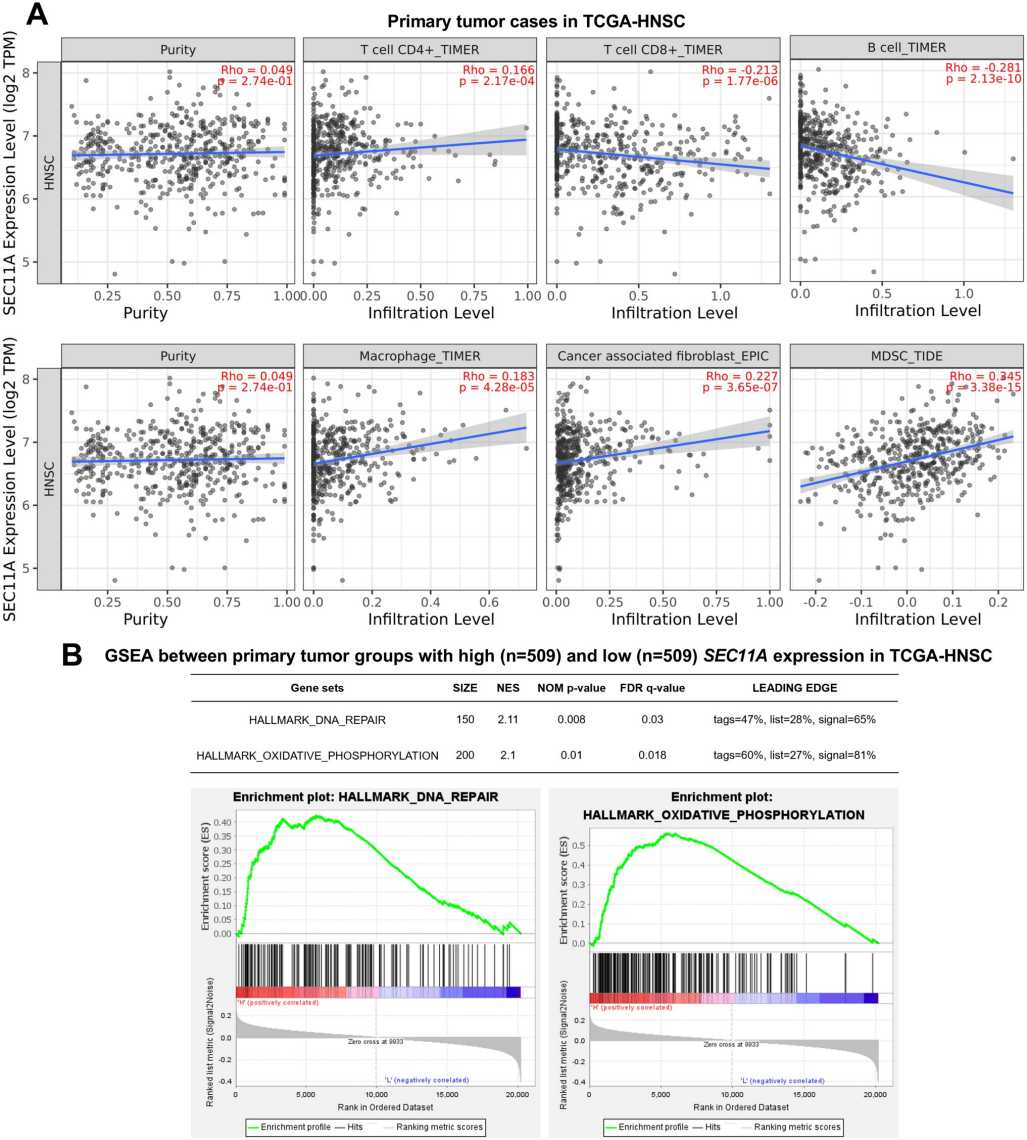
*SEC11A* expression might predict the infiltration of immune cells in the tumor microenvironment. **A.** Pearson’s rho values were calculated to assess the correlation between the expression of SEC11A and the infiltration of immune cells in the head and neck squamous cell carcinoma associated tumor microenvironment, including CD4+ T cells, CD8+ T cells, B cells, macrophages, cancer-associated fibroblasts and MDSCs. **B.** A summary (top) and representative images (bottom) of plot images of the gene sets significantly enriched in the high *SEC11A* expression group.

Using immune cell estimation provided by Tumor Immune Estimation Resource 2.0 (TIMER 2.0) (http://timer.cistrome.org/) [16], we assessed the correlation between SEC11A expression and the abundance of immune cells in the tumor microenvironment. By setting r>|0.2| as the cutoff, we found that *SEC11A* expression was negatively correlated with CD8+ T cells and B cells, but was positively correlated with cancer-associated fibroblast and myeloid-derived suppressor cells (MDSCs) (Fig 4A). Then, we conducted GSEA to explore the dysregulated gene sets between high and low *SEC11A* expression groups. Results showed that the high *SEC11A* expression group had higher expression of the genes enriched in DNA repair and oxidative phosphorylation (Fig 4B).

## Discussion

In this study, we examined the expression of the five SPC subunit genes and their prognostic value in head and neck squamous cell carcinoma. Among the five genes, *SEC11A* upregulation can serve as an independent prognostic biomarker regarding PFS and DSS. In addition, our subgroup analysis confirmed the prognostic value in tumors originating from different anatomic sites.

*SEC11A* can promote the phosphorylation of EGFR and the downstream kinases, including ERK and Akt (3). This association was confirmed in tongue squamous cell carcinoma, a major subtype of head and neck squamous cell carcinoma [6]. EGFR is overexpressed in approximately 50 to 90% of head and neck squamous cell carcinoma cases [17, 18]. Its activation results in a phosphorylation cascade involving a series of downstream signaling effectors, such as PI3K-AKT, MAPK, ERK, and Jak/STAT. Activation of these signaling pathways can enhance the growth, invasion, angiogenesis, and metastasis of the tumor [19–21]. EFGR can be overactivated via multiple machinimas, such as overexpression of EGFR and its ligands, genetic mutations/polymorphism and activation by other receptor tyrosine kinases (RTKs) [18–20]. In addition, activation of the EGFR signaling pathway can induce resistance to chemo-radiation in cancers by promoting DNA repair via multiple mechanisms, such as activating DNA dependent protein kinase, promoting the transcription of DNA repair genes (*RAD51*, *ATM*, and *XRCC1*) and phosphorylating proliferating cell nuclear antigen (PCNA) [22]. This mechanism helps explain why SEC11A upregulation is associated with increased genes enriched in the DNA repair set in our GSEA. Therefore, *SEC11A* upregulation might serve as an important mechanism for overactivated EGFR. Due to its critical regulative effects on cancer cell behaviors, EGFR has been considered a promising therapeutic target. Currently, the EGFR monoclonal antibody cetuximab is generally administrated in combination with radiation for patients with HPV-negative head and neck squamous cell carcinoma [23]. To Understand the mechanisms underlying *SEC11A* upregulation, we checked its DNA methylation status and gene-level copy numbers. Our analysis showed that *SEC11A* upregulation might not be regulated by CpG methylation within its gene locus, but is associated with its gene-level amplification.

Using data from one previous single-cell RNA-seq database, we found that SEC11A is expressed not only in tumor cells, but also in other cells in the tumor microenvironment. Therefore, we hypothesized that SEC11A might also modulate the malignant behaviors of head and neck squamous cell carcinoma via other mechanisms. By checking immune cell abundance in the tumor microenvironment, we observed a significant positive correlation between SEC11A expression and the infiltration of MDSCs, a group of immature myeloid cells with immunosuppressive effects [24]. Previous studies showed that the abundance of MDSCs is associated with advanced stages of head and neck squamous cell carcinoma and poor prognosis [25, 26]. By conducting GSEA, we observed that the high *SEC11A* expression group had increased the expression of genes enriched in the oxidative phosphorylation set. Tumor-infiltrating MDSCs have increased fatty acid uptake, oxidation, and oxidative phosphorylation [27]. These alterations were associated with increased mitochondrial mass, upregulation of the key fatty acid oxidation enzymes, and an elevated oxygen consumption rate [28]. Therefore, we infer that SEC11A associated elevation of the oxidative phosphorylation genes might generate a favorable tumor microenvironment for the infiltration or conversion of MDSCs.

This study also has some limitations. Firstly, since we did not find other databases with large samples, detailed anatomic information and survival data together, no validation cohort was used to verify the prognostic significance of *SEC11A* expression. Secondly, the molecular mechanisms underlying the association among *SEC11A* expression, the elevation of the oxidative phosphorylation genes and the infiltration or conversion of MDSCs were not explored in the current study. Future studies are warranted for a better understanding of the regulatory mechanisms of SEC11A.

## Conclusion

In this study, we revealed that *SEC11A* upregulation is a result of gene amplification in head and neck squamous cell carcinoma. Its upregulation serves as an independent prognostic biomarker regarding PFS and DSS. Besides, *SEC11A* expression might predict the infiltration of certain types of immune cells in the tumor microenvironment.

## Supporting information

S1 Fig Subgroup analysis of PFS and DSS in HPV16 positive or negative head and neck squamous cell carcinoma. **A-D.** K-M survival analysis was performed to compare the differences in PFS (A-B) and DSS (C-D) in patients grouped by known HPV16 infection status. Log-rank test was used to compare the curves and calculate the *p* values.
(TIF)

S1 Checklist *PLOS ONE* clinical studies checklist. (PDF)

## References

[1] Liaci AM, Steigenberger B, Telles de Souza PC, Tamara S, Grollers-Mulderij M, Ogrissek P, et al. Structure of the human signal peptidase complex reveals the determinants for signal peptide cleavage. Mol Cell. 2021;81(19):3934–48 e11. Epub 2021/08/14. doi: 10.1016/j.molcel.2021.07.031 . 34388369

[2] Oue N, Naito Y, Hayashi T, Takigahira M, Kawano-Nagatsuma A, Sentani K, et al. Signal peptidase complex 18, encoded by SEC11A, contributes to progression via TGF-alpha secretion in gastric cancer. Oncogene. 2014;33(30):3918–26. Epub 2013/09/03. doi: 10.1038/onc.2013.364 . 23995782

[3] Hattori T, Sentani K, Naohide O, Sakamoto N, Yasui W. Clinicopathological significance of SPC18 in colorectal cancer: SPC18 participates in tumor progression. Cancer Sci. 2017;108(1):143–50. Epub 2016/11/20. doi: 10.1111/cas.13121 27859949 PMC5276824

[4] Shigematsu Y, Oue N, Sekino Y, Sakamoto N, Sentani K, Uraoka N, et al. SEC11A Expression Is Associated with Basal-Like Bladder Cancer and Predicts Patient Survival. Pathobiology. 2019;86(4):208–16. Epub 2019/06/05. doi: 10.1159/000497206 . 31163419

[5] Yamamoto Y, Oue N, Asai R, Katsuya N, Uraoka N, Sakamoto N, et al. SPC18 Expression Is an Independent Prognostic Indicator of Patients with Esophageal Squamous Cell Carcinoma. Pathobiology. 2020;87(4):254–61. Epub 2020/06/22. doi: 10.1159/000506956 . 32564026

[6] Yao Y, Liu XQ, Yang FY, Mu JW. MiR-873-5p modulates progression of tongue squamous cell carcinoma via targeting SEC11 A. Oral Dis. 2021. Epub 2021/03/07. doi: 10.1111/odi.13830 . 33675129

[7] Hwang S, Mahadevan S, Qadir F, Hutchison IL, Costea DE, Neppelberg E, et al. Identification of FOXM1-induced epigenetic markers for head and neck squamous cell carcinomas. Cancer. 2013;119(24):4249–58. Epub 2013/10/12. doi: 10.1002/cncr.28354 . 24114764

[8] Wang X, Zhou X, Liu J, Liu Z, Zhang L, Gong Y, et al. Genomewide investigation of the clinical implications and molecular mechanism of long noncoding RNA LINC00668 and proteincoding genes in hepatocellular carcinoma. Int J Oncol. 2019;55(4):860–78. Epub 2019/08/23. doi: 10.3892/ijo.2019.4858 31432149 PMC6741837

[9] Wang X, Dou X, Ren X, Rong Z, Sun L, Deng Y, et al. A Ductal-Cell-Related Risk Model Integrating Single-Cell and Bulk Sequencing Data Predicts the Prognosis of Patients With Pancreatic Adenocarcinoma. Front Genet. 2021;12:763636. Epub 2022/01/21. doi: 10.3389/fgene.2021.763636 35047000 PMC8762279

[10] Cristina V, Herrera-Gomez RG, Szturz P, Espeli V, Siano M. Immunotherapies and Future Combination Strategies for Head and Neck Squamous Cell Carcinoma. Int J Mol Sci. 2019;20(21). Epub 2019/11/02. doi: 10.3390/ijms20215399 31671550 PMC6862353

[11] Goldman MJ, Craft B, Hastie M, Repecka K, McDade F, Kamath A, et al. Visualizing and interpreting cancer genomics data via the Xena platform. Nat Biotechnol. 2020;38(6):675–8. Epub 2020/05/24. doi: 10.1038/s41587-020-0546-8 . 32444850 PMC7386072

[12] Gu Y, Hu C. Bioinformatic analysis of the prognostic value and potential regulatory network of FOXF1 in papillary thyroid cancer. Biofactors. 2019;45(6):902–11. Epub 2019/09/10. doi: 10.1002/biof.1561 . 31498939

[13] Puram SV, Tirosh I, Parikh AS, Patel AP, Yizhak K, Gillespie S, et al. Single-Cell Transcriptomic Analysis of Primary and Metastatic Tumor Ecosystems in Head and Neck Cancer. Cell. 2017;171(7):1611–24 e24. Epub 2017/12/05. doi: 10.1016/j.cell.2017.10.044 29198524 PMC5878932

[14] Sun Y, Pan H, He Y, Hu C, Gu Y. Functional roles of the SHCBP1 and KIF23 interaction in modulating the cell-cycle and cisplatin resistance of head and neck squamous cell carcinoma. Head Neck. 2021. Epub 2021/12/18. doi: 10.1002/hed.26961 . 34918847

[15] Fleming JC, Woo J, Moutasim K, Mellone M, Frampton SJ, Mead A, et al. HPV, tumour metabolism and novel target identification in head and neck squamous cell carcinoma. Br J Cancer. 2019;120(3):356–67. Epub 2019/01/19. doi: 10.1038/s41416-018-0364-7 30655616 PMC6353968

[16] Li T, Fu J, Zeng Z, Cohen D, Li J, Chen Q, et al. TIMER2.0 for analysis of tumor-infiltrating immune cells. Nucleic Acids Res. 2020;48(W1):W509–W14. Epub 2020/05/23. doi: 10.1093/nar/gkaa407 32442275 PMC7319575

[17] Dubot C, Bernard V, Sablin MP, Vacher S, Chemlali W, Schnitzler A, et al. Comprehensive genomic profiling of head and neck squamous cell carcinoma reveals FGFR1 amplifications and tumour genomic alterations burden as prognostic biomarkers of survival. Eur J Cancer. 2018;91:47–55. Epub 2018/01/15. doi: 10.1016/j.ejca.2017.12.016 . 29331751

[18] Bossi P, Resteghini C, Paielli N, Licitra L, Pilotti S, Perrone F. Prognostic and predictive value of EGFR in head and neck squamous cell carcinoma. Oncotarget. 2016;7(45):74362–79. Epub 2016/08/25. doi: 10.18632/oncotarget.11413 27556186 PMC5342059

[19] Marquard FE, Jucker M. PI3K/AKT/mTOR signaling as a molecular target in head and neck cancer. Biochem Pharmacol. 2019;172:113729. Epub 2019/12/01. doi: 10.1016/j.bcp.2019.113729 . 31785230

[20] Cancer Genome Atlas N. Comprehensive genomic characterization of head and neck squamous cell carcinomas. Nature. 2015;517(7536):576–82. Epub 2015/01/30. doi: 10.1038/nature14129 25631445 PMC4311405

[21] Yao Y, Liu Z, Zhao M, Chen Z, Li P, Zhang Y, et al. Design, synthesis and pharmacological evaluation of 4-(3-chloro-4-(3-cyclopropylthioureido)-2-fluorophenoxy)-7-methoxyquinoline-6-car boxamide (WXFL-152): a novel triple angiokinase inhibitor for cancer therapy. Acta Pharm Sin B. 2020;10(8):1453–75. Epub 2020/09/24. doi: 10.1016/j.apsb.2020.04.002 32963943 PMC7488503

[22] Cuneo KC, Nyati MK, Ray D, Lawrence TS. EGFR targeted therapies and radiation: Optimizing efficacy by appropriate drug scheduling and patient selection. Pharmacol Ther. 2015;154:67–77. Epub 2015/07/25. doi: 10.1016/j.pharmthera.2015.07.002 26205191 PMC4570853

[23] Johnson DE, Burtness B, Leemans CR, Lui VWY, Bauman JE, Grandis JR. Head and neck squamous cell carcinoma. Nat Rev Dis Primers. 2020;6(1):92. Epub 2020/11/28. doi: 10.1038/s41572-020-00224-3 . 33243986 PMC7944998

[24] Li K, Shi H, Zhang B, Ou X, Ma Q, Chen Y, et al. Myeloid-derived suppressor cells as immunosuppressive regulators and therapeutic targets in cancer. Signal Transduction and Targeted Therapy. 2021;6(1):362. doi: 10.1038/s41392-021-00670-9 34620838 PMC8497485

[25] Chen WC, Lai CH, Chuang HC, Lin PY, Chen MF. Inflammation-induced myeloid-derived suppressor cells associated with squamous cell carcinoma of the head and neck. Head Neck. 2017;39(2):347–55. Epub 2016/10/04. doi: 10.1002/hed.24595 . 27696591

[26] Peltanova B, Raudenska M, Masarik M. Effect of tumor microenvironment on pathogenesis of the head and neck squamous cell carcinoma: a systematic review. Mol Cancer. 2019;18(1):63. Epub 2019/04/01. doi: 10.1186/s12943-019-0983-5 30927923 PMC6441173

[27] Yan D, Adeshakin AO, Xu M, Afolabi LO, Zhang G, Chen YH, et al. Lipid Metabolic Pathways Confer the Immunosuppressive Function of Myeloid-Derived Suppressor Cells in Tumor. Front Immunol. 2019;10:1399. Epub 2019/07/06. doi: 10.3389/fimmu.2019.01399 31275326 PMC6593140

[28] Kumar V, Patel S, Tcyganov E, Gabrilovich DI. The Nature of Myeloid-Derived Suppressor Cells in the Tumor Microenvironment. Trends Immunol. 2016;37(3):208–20. Epub 2016/02/10. doi: 10.1016/j.it.2016.01.004 26858199 PMC4775398

