# Supplementary figures and images for: Signal peptidase complex catalytic subunit SEC11A upregulation is a biomarker of poor prognosis in patients with head and neck squamous cell carcinoma
Source: PLoS One. 2022 Jun 2;17(6):e0269166. doi: 10.1371/journal.pone.0269166 (PMC9162331; doi:10.1371/journal.pone.0269166)

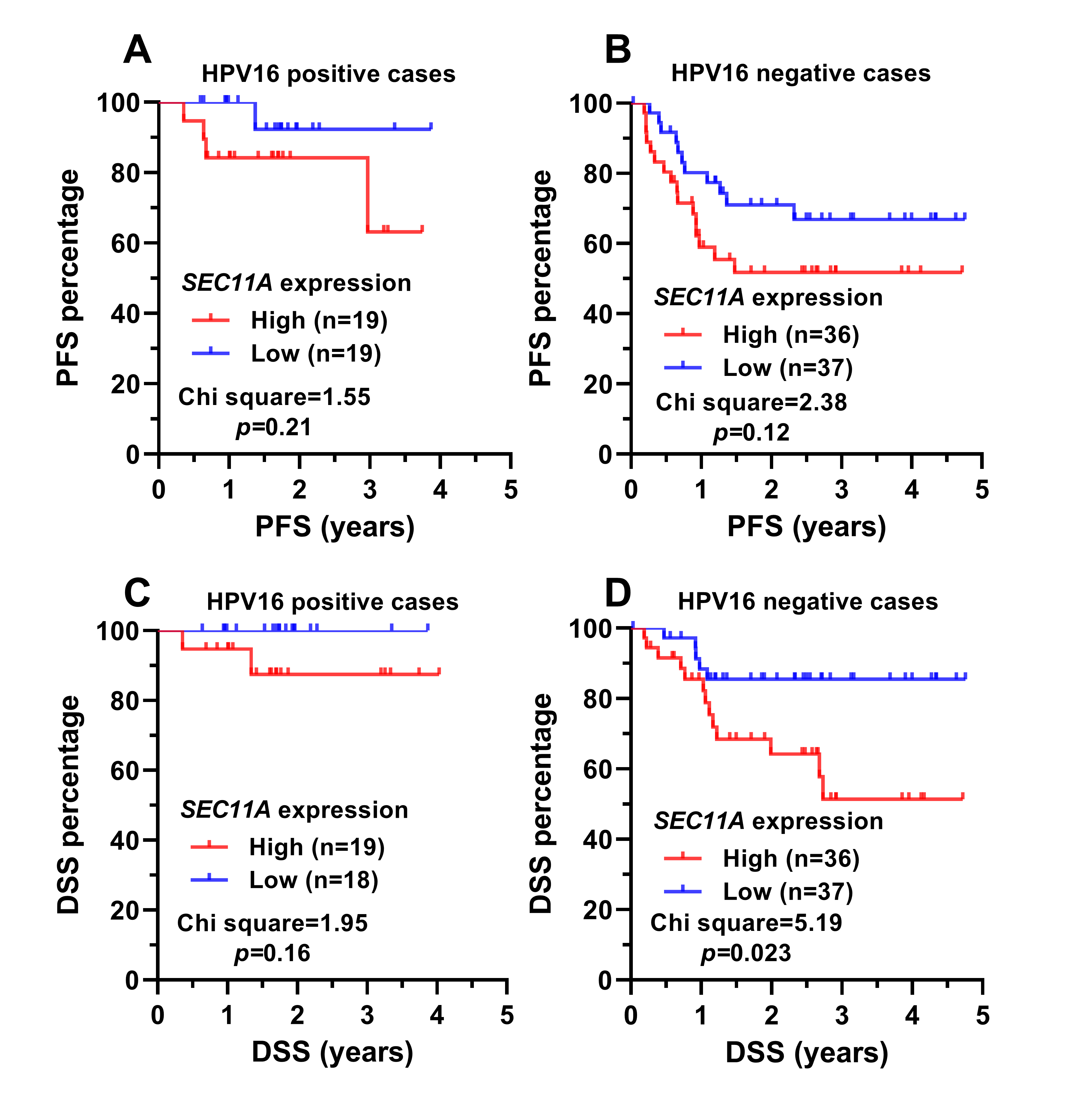

Supplement: S1 Fig — A-D. K-M survival analysis was performed to compare the differences in PFS (A-B) and DSS (C-D) in patients grouped by known HPV16 infection status. Log-rank test was used to compare the curves and calculate the p values. (TIF) [file pone.0269166.s001.tif]
